# Neural correlates of conversion disorder: overview and meta-analysis of neuroimaging studies on motor conversion disorder

**DOI:** 10.1186/s12888-016-0890-x

**Published:** 2016-06-10

**Authors:** Markus Boeckle, Gregor Liegl, Robert Jank, Christoph Pieh

**Affiliations:** Department of Psychotherapy and Biopsychosocial Health, Danube University Krems, Dr.-Karl-Dorrek-Str. 30, 3500 Krems, Austria; Department of Cognitive Biology, University of Vienna, Vienna, Austria; Medical Clinic, Department of Psychosomatic Medicine, Charité - Universitätsmedizin, Berlin, Germany; Department of Psychosomatic Medicine, University Hospital Regensburg, Regensburg, Germany

**Keywords:** Motor conversion disorder, Hysteria, Meta-analysis, Neuroimaging, Neurology

## Abstract

**Background:**

Conversion Disorders (CD) are prevalent functional disorders. Although the pathogenesis is still not completely understood, an interaction of genetic, neurobiological, and psychosocial factors is quite likely. The aim of this study is to provide a systematic overview on imaging studies on CDs and investigate neuronal areas involved in Motor Conversion Disorders (MCD).

**Methods:**

A systematic literature search was conducted on CD. Subsequently a meta-analysis of functional neuroimaging studies on MCD was implemented using an Activation Likelihood Estimation (ALE). We calculated differences between patients and healthy controls as well as between affected versus unaffected sides in addition to an overall analysis in order to identify neuronal areas related to MCD.

**Results:**

Patients with MCD differ from healthy controls in the amygdala, superior temporal lobe, retrosplenial area, primary motor cortex, insula, red nucleus, thalamus, anterior as well as dorsolateral prefrontal and frontal cortex. When comparing affected versus unaffected sides, temporal cortex, dorsal anterior cingulate cortex, supramarginal gyrus, dorsal temporal lobe, anterior insula, primary somatosensory cortex, superior frontal gyrus and anterior prefrontal as well as frontal cortex show significant differences.

**Conclusions:**

Neuronal areas seem to be involved in the pathogenesis, maintenance or as a result of MCD. Areas that are important for motor-planning, motor-selection or autonomic response seem to be especially relevant. Our results support the emotional unawareness theory but also underline the need of more support by conduction imaging studies on both CD and MCD.

## Background

In the recent Diagnostic and Statistical Manual of Mental Disorders (DSM-5; [1]), conversion disorder (CD) is defined as (1) having at least one symptom of altered voluntary motor or sensory function, (2) the presence of clinical findings supporting incompatibility between symptom and neurological or medical conditions, (3) the symptom is not better explained by another medical or mental disorder, and (4) causes clinically significant distress or impairment. Due to problems of case definition and case ascertainment, prevalence rates vary largely [2]. However, CDs are not rare conditions, with prevalence rates of 1 to 3 % in the general population [3, 4]. A prospective cohort study found that 5.6 % of all outpatients have CD [5] and we suppose that treatment of patients with CD might be highly demanding similar to the treatment of patients with somatoform disorders [6].

Despite the historical relevance of the disorder in relation to hysteria, the current knowledge on aetiology and neurological background of CD is incomplete [7]. Similar to other psychiatric disorders an interrelation of genetic, neurobiological, and psychosocial factors is highly plausible. Twin studies showed that approximately 50 % of the variance could be explained by genetic factors [8]. Dissociative symptoms are reported as a side effect of medication [9] and associated with endocrinological disorders [10], which points to neurobiological influences [7, 11]. Furthermore, psychosocial influences are assumed in the pathogenesis of dissociative disorders, which are according to ICD-10 [12] closely related to CD. Psychological factors like alexithymia - the inability to identify and describe emotions in the self - is a risk factor for dissociative disorders [13]. There is rising evidence that dissociative symptoms are associated with trauma, as depersonalization and derealisation are quintessential responses to acute trauma [14] and dissociative symptoms often occur in patients with post-traumatic stress disorder (PTSD) [15].

Due to the relatedness of these disorders, similarities in the pathology might occur. For motor and somatosensory conversion Perez and colleagues [7] discuss different explanatory models of brain function: (1) disrupted inhibitory abilities with dysfunctionality in primary somatosensory and motor cortex [16–19] (2) modifications of the voluntary-intentional capacities with dysfunctionality in prefrontal areas [20, 21] (3) impaired attention based on dysfunctional anterior cingulate cortex, parietal associative cortex, striatum, thalamus [18, 22–24] (4) misconceptions of action authorship as a result of dysfunctionality in the tempoparietal junction, somatosensory cortex, anterior cingulate cortex, parietal associative cortex, and gyrus temporalis superior [25] (5) as well as affective disorders due to dysfunctionality in the amygdala and anterior cingulate cortex [25–27]. In addition to functional differences, structural changes have recently been discussed in conjunction with CD [28, 29] whereby the premotor cortex, the primary motor cortex, and the cerebellum show changes in cortical thickness.

Although there is an incremental increase of knowledge about the causes of dissociative disorders, there is currently a lack of evidence for the effectiveness of psychological and pharmacological treatments. The International Society for the Study of Trauma and Dissociation noted in its guidelines that the treatment of Dissociative Identity Disorder is still in its infancy [14, 30]. For this reason, a recent study recommends further neurophysiological studies, including fMRI studies [31]. These studies should aim to not only provide more information about the aetiology of dissociative disorders, but also to identify at risk patients in a more timely manner and to thus treat dissociative conditions early and appropriately [31].

Even though numerous neuroimaging studies have been conducted on CD (e.g.: [2, 11, 32]) and neurobiological models have been proposed [7, 33], a meta-analytical approach is still missing. This might be due to the fact that different dissociative disorders, such as dissociative amnesia, fugue, dissociative identity disorder, motor and somatosensory conversions as well as pseudo-epileptic seizures might have various neurobiological correlates. We therefore provide as a first step a detailed list of publication on CD identified via a systematic literature research. However, because of potential differences in neurobiology between these disorders we mainly focused on motor conversion disorder (MCD) by conducting as a second step a meta-analytic approach on MCD using an activation likelihood estimation (ALE) to investigate neurobiological correlates of MCD.

## Methods

### Literature search

In order to identify the research articles relating to CD that utilized neuro-imaging methods, we searched the following scientific databases: Medline, Psycinfo, Psyndex, and Cochrane. We did so using the following search terms: *(“dissociative disorder” OR “functional disorder” OR “conversion disorder”)*, which simultaneously included the following neuro-imaging methods: *(“neuro imaging” OR (“magnetic resonance imaging” OR (“magnetic” AND “resonance” AND “imaging”) OR “magnetic resonance imaging” OR “fMRI”) OR (“magnetic resonance imaging” OR (“magnetic” AND “resonance” AND “imaging”) OR “magnetic resonance imaging” OR “MRI”) OR VBM OR PET)*. We included all published articles until August 2015. All titles and abstracts were independently rated by MB, GL, and RJ. All articles identified to include all given search terms by at least one of the raters were included in a subsequent full-text analysis. The criteria for inclusion in both steps (abstract and title analysis) were identical. The inclusion criteria were defined as the following: 1) paper written in English; 2) investigating human adults; 3) has to be primary research (thus excluding editorials, letters to the editor, systematic reviews, case studies, etc.); 4) study has to use one of the listed imaging methods (PET, MRI, SPECT); 5) studies investigated patients with CD or synonymous disorder according to DSM-IV, DSM-V, or ICD-10 specifically excluding studies using hypnosis or feigning behaviour as alternative study population for CD. Any matches were included in the subsequent full text analysis process. All remaining articles were checked for accordance to the inclusion criteria. After analysing the full texts, we reported all studies using neuroimaging and CD or dissociative disorders as the classification of CD in ICD and DSM differ according to this point. As a next step we identified all studies about MCD that included imaging methods. For the inclusion in the meta-analysis, both neuro-imaging and MCD criterion had to be met and coordinates in MNI or Talairach space had to be provided.

### Meta-analysis

We conducted an Activation Likelihood Estimation (ALE) using GingerALE 2.3.1 [34–36], which supports the integration of multiple neuroimaging studies across imaging methods. Imaging studies based on MRI, PET, or SPECT and reporting Talairach or MNI coordinates including patients with MCD were included in the analysis. Coordinates published in MNI space were transformed into Talairach space using icbm2tal transformation [37, 38] provided by brainmap.org [39]. We incorporated all significant differences listed in the included papers. We used a Cluster-Level Analysis when analysing all experiments to correct for multiple identifications within one experiment as this procedure accommodates the spatially contiguous nature of the signal [36] and allows comparisons across different cognitive processes [35]. For the correction of multiple comparisons we used an uncorrected p-value of 0.001 as the cluster-forming threshold [40, 41], a cluster-level inference level of 0.05 with 1000 permutations, and a minimum cluster volume of 264 mm^2^. Threshold maps were viewed with Mango version 3.8 (772) [42], and significant clusters from the ALE analysis were superimposed on a standard anatomical image of the entire brain (Colin1.1.nii).

We analysed functional alterations in relation to MCD between patients and healthy controls for increased and decreased activation in patients separately. We calcualted additional subgroup meta-analyses in order to differentiate between increased and decreased activations of the affected versus unaffected sides of the brain. We excluded studies investigating the difference between dissociative disorders and hypnotized controls because of possible differences in neurobiological correlates.

The first analysis combined all reported functional imaging experiments within 12 articles. It includes 187 subjects, 73 foci and nine foci lying outside of the mask. In the subset analysis, in which greater activity in MCD patients versus healthy controls was analysed (p>c), is comprised of 148 subjects with 31 foci out of 6 studies, whereby four foci were lying outside of the mask. The sub analysis of patients showing lower activation than healthy controls (p<c) is based on four foci from two experiments and 36 individuals. The analysis of affected sides showing increased activation than the unaffected (a>ua) side and vice versa (a<ua) is based on 27 foci from 4 experiments with 28 individuals and six foci from two experiments with 13 individual respectively. Low numbers of foci located outside the mask do not influence the results of the ALE analysis. Grey matters are reported within the nearest +/− 1 mm. When recording the number of subjects in articles with multiple experiments, we used the lowest number of subjects reported in the respective article in order to calculate the most conservative ALE possible. Thus, when including the same study in different subsamples, samples from the same article can show varying numbers of subjects, which we statistically accounted for via cluster level analysis. Articles might report differing numbers of subjects dependent on the specific tests. We therefore used the lowest reported number of subjects that was listed for relevant subtests of one article, in order to calculate the most conservative influence of these areas. We used cluster-level analysis in order to adjust for the overlap of subjects reported from multiple tasks within one study.

## Results

### Study selection

**Fig. 1:**
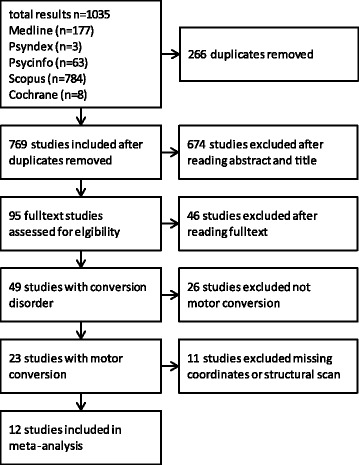
Flow chart of systematic literature review process

The systematic literature search yielded 1035 results, whereby 266 duplicates were excluded; resulting in a total of 769 studies (Fig. 1). After a thorough analysis of the abstracts and titles (which led to the exclusion of 674 studies), 95 full texts were assessed to be potentially eligible for inclusion in this study. After full text analysis, the search resulted in 49 studies with neuroimaging of CD (Table 1).

**Table 1:** All studies found with conversion disorder and neuroimaging. Studies indicated with ^a^ were included in the meta-analysis

Study	Disorder	Control group	Number of participants	Imaging method
Atmaca, et al. [82]	motor conversion	yes	patients: 12 (f), control: 12 (f)	sMRI (1.5 Tesla)
Atmaca, et al. [83]	somatization disorder	yes	patients: 20 (f), control: 20 (f)	sMRI (1.5 Tesla)
Atmaca, et al. [84]	motor conversion	yes	patients: 20 (f), control: 20 (f)	sMRI (1.5 Tesla)
Aybek, et al. [29]	motor conversion	yes	patients: 15 (11f, 4m) [2 groups: hemiparesis & paraparesis], control: 25 (16f, 9m)	sMRI (3.0 Tesla)
^a^Aybek, et al. [52]	motor conversion	yes	patients: 12 (9f, 3m), control 14 (11f, 3m)	fMRI (3.0 Tesla)
^a^Aybek, et al. [47]	motor conversion	yes	patients: 12 (8f, 4m), control 13 (10f, 3m)	fMRI (3.0 Tesla)
Benbadis, et al. [85]	syncope of unknown origin	no	patients: 10 (5f, 5m)	CT (7 patients), MRI (8 patients)
Blakemore, et al. [86]	motor conversion	yes	patients: 6 (4f, 2m), feigner: 12 (8f), control: 12 (8f)	EEG
Blakemore, et al. [87]	motor conversion	yes	patients: 6 (4f, 2m), feigner: 12 (8f), control: 12 (8f)	EEG
Bonilha, et al. [88]	idiopathic dystonia	yes	patients: 7 (6f, 1m), control: 10 (8f, 2m)	sMRI (3.0 Tesla)
Burke, et al. [89]	sensory conversion	no	Patients: 10 (10f)	fMRI (3.0 Tesla)
Burgmer, et al. [90]	motor conversion	yes	patients: 4 (m), control: 7 (3f, 4m)	fMRI (3.0 Tesla)
Carey, et al. [91]	body dysmorphic disorder	no	patients 6 (4m, 2f)	SPECT (HMPAO)
Cojan, et al. [19]	motor conversion	yes	patients: 1 (f), control: 30 (normal:24, feigner: 6)	fMRI (1.5 Tesla)
^a^Czarnecki, et al. [60]	motor conversion	yes	patients:5 (3f, 2m)	SPECT (99mTc-Ethyl cysteinate)
^a^de Lange, et al. [16]	motor conversion	no	patients: 8 (5f, 3m)	fMRI (1.5 Tesla)
^a^de Lange, et al. [64]	motor conversion	no	patients:7 (5f, 2m)	fMRI (1.5 Tesla)
de Lange, et al. [21]	motor conversion	no	patients: 8	fMRI (1.5 Tesla)
de Ruiter, et al. [92]	non clinical dissociative experiences	yes	individuals: 43 (23 low (15f, 8m), 20 high (10f, 10m)	fMRI (1.5 Tesla)
Devinsky, et al. [93]	C-NES	yes	C-NES only:22, C-NES + Epilepsy:38, Epilepsy only:43,Epilepsy + other psych:59	EEG
^a^Elzinga, et al. [46]	motor conversion	yes	patients: 13 (f), control:14 (f)	fMRI (1.5 Tesla)
Felmingham, et al. [94]	dissociative PTSD	yes	patients:23 (13f, 10m), 12 dissociative	fMRI (1.5 Tesla)
Feusner, et al. [95]	body dysmorphic disorder	yes	patients: 12 (10f, 2m), control: 13 (11f, 2m)	fMRI (3.0 Tesla)
Feusner, et al. [96]	body dysmorphic disorder	yes	patients: 12 (10f, 2m), control: 12 (10f, 2m)	sMRI (3.0 Tesla)
Garcia-Campayo, et al. [97]	somatization disorder	no	patients: 11 (5f, 6m)	SPECT (HMPAO or TC-bicisate)
Ghaffar, et al. [62]	motor conversion	yes	patients: 3 (3f), control: 6	fMRI (3.0 Tesla)
Hakala, et al. [98]	somatization disorder	yes	patients: 10 (10f), control: 16 (16f)	sMRI (1.5 Tesla)
Hoechstetter, et al. [99]	motor conversion	no	patients: 3 (2f, 1m)	MEG
Hovorka, et al. [100]	PNES	no	patients: 56 (39f, 17m)	EEG
Karatas, et al. [101]	PNES	no	patients: (88)	EEG
Knyazeva, et al. [102]	PNES	yes	patients: 13 (8f, 5m), control: 13 (8f, 5m)	EEG
Krüger, et al. [103]	dissociation DES	yes	patients: 50	EEG
Labate, et al. [28]	PNES	yes	patients: 20 (11f, 9m), control: 40 (21f, 19m)	sMRI (1.5 Tesla)
Mailis-Gagnon, et al. [24]	hysterical anaesthesia	no	patients: 4 (3f, 1m)	fMRI (1.5 Tesla)
Moser, et al. [104]	dissociation	yes	patients: 11 (11f), control: 9 (9f)	fMRI (3.0 Tesla)
Nicholson, et al. [105]	motor conversion	yes	patients: 15 (10f, 5m) control: 31 (19f, 12m)	sMRI (3.0 Tesla)
Rauch, et al. [106]	body dysmorphic disorder	yes	patients: 8 (?) control: 8 (?)	MRI
Roelofs, et al. [66]	motor conversion	no	patients: 6 (f)	EEG
Sar, et al. [107]	dissociative identity disorder	yes	patients: 21 (14f, 7m), control: 9 (6f, 3m)	SPECT (HMPAO)
^a^Spence, et al. [20]	motor conversion	yes	patients: 2 (m), control: 6	PET
^a^Stone, et al. [74]	motor conversion	yes	patients: 4 (3f, 1m), control: 4 (3f, 1m)	fMRI (1.5 Tesla)
^a^van Beilen, et al. [50]	motor conversion	yes	patients: 9 (7f, 2m), control: 21 normal control (17f, 4m) 13 feigning (4f, 9m)	fMRI (3.0 Tesla)
van Der Kruijs, et al. [108]	PNES	yes	patients: 11 (6f, 5m), control: 12 (8f, 4m)	fMRI (3.0 Tesla)
^a^Voon, et al. [43]	conversion tremor, dystonia, gait disorder	yes	patients: 11 (7f, 4m), control: 11 (7f, 4m)	fMRI (1.5 Tesla)
^a^Voon, et al. [27]	motor conversion	yes	patients: 16 (10f, 6m), control: 16 (10f, 6m)	fMRI (1.5 Tesla)
Voon, et al. [25]	motor conversion	no	patients: 8 (5f, 3m)	fMRI (1.5 Tesla)
^a^Vuilleumier, et al. [22]	sensorimotor conversion	no	patients: 7 (6f, 1m)	PET (HMPAO)
Werring, et al. [109]	sensory conversion	yes	patients: 5 (4f, 1m), control 7 ()	fMRI (1.5 Tesla)
Yazici, et al. [110]	Astasia-Abasia	no	patients: 5 (3f, 2m)	PET (HMPAO)

*Abbreviations*: *CT* X-ray computed tomography; *EEG* electroencephalography; *fMRI* functional magnetic resonance tomography; *sMRI* structural magnetic resonance tomography; *MEG* magnetoencephalography; *SPECT* single-photon emission computed tomography; *PET* positron emission tomography, sex of individuals is indicated in brackets when reported in the study, ^a^indicates studies that were included in the meta-analysis

### Study characteristics

The systematic literature retrieval with the applied search terms came forth with 49 studies with neuroimaging of CD: 23 studies about MCD, 5 about psychogenic non-epileptic seizures, 4 about body dysmorphic disorder, 3 about somatization disorder, 3 about pure sensory conversion, and 11 studies about single disorders (for details see Table 1). All 49 studies either used MRI, CT, Spect, EEG, MEG, or PET as their imaging method. Out of all studies, 34 used a control group, whereby the others used within-subject differences (Table 1). Of these 49 studies, 26 studies were excluded from the meta-analysis because they were not about MCD, 10 studies because of missing coordinates. We additionally excluded one study conducting structural scans, which resulted in 12 studies considered for meta-analyses. Out of the 23 included studies reporting imaging results of MCD, 16 studies used a control group (Table 2). Three studies looked at differences between affected and non-affected sides of the body (Table 2). All studies showed difference in (Table 2). The 12 studies that included MNI or Talairach coordinates, reported different affected areas listed in Table 3.

**Table 2:** Neuroimaging studies on motor conversion disorders

Citation	Type of conversion	Study design	Task
Atmaca, et al. [82]	unilateral motor symptoms	matched control (healthy control)	structural differences
Aybek, et al. [29]	conversion disorder with limb weakness	matched control (healthy control)	structural differences
Aybek, et al. [52]	motor conversion disorder	matched control (healthy control)	visual stimuli - emotional faces
Aybek, et al. [47]	motor conversion disorder	matched control (healthy control)	stressful memories task
Blakemore, et al. [86]	unilateral upper limb conversion paresis	matched control (healthy control)	visual stimuli - reaction time task
Burgmer, et al. [90]	dissociative paralysis in conversion disorders	matched controls	movement execution and observation-task
Burke, et al. [89]	unilateral conversion disorder, sensory subtype	within group differences	vibrotactil stimulation
Czarnecki, et al. [60]	psychogenic movement disorders	matched control (healthy control)	resting state
			simple motor task
			task vs. rest
de Lange, et al. [16]	conversion paralysis	within subjects design	motor imagery task
de Lange, et al. [64]	full or partial paralysis lateralized to one arm	within subjects design	motor imagery task
de Lange, et al. [21]	full or partial conversion paralysis lateralized to one hand	within subject design	motor imagery task
Elzinga, et al. [46]	dissociative disorder	matched control (healthy control)	working memory task effects
			task load
Ghaffar, et al. [62]	sensorimotor loss	case series	vibratory stimulation
Nicholson, et al. [105]	motor conversion	matched control (healthy control)	structural
Roelofs, et al. [66]	conversion paralysis	within subjects design	two-choice reaction task
Spence, et al. [20]	motor conversion	matched control (healthy control)	movement execution
Stone, et al. [74]	motor conversion	matched control (healthy control)	movement execution
van Beilen, et al. [50]	motor conversion (paresis)	Within subjects design, matched control (healthy control)	movement execution and imagination
Voon, et al. [27]	conversion disorder with positive motor symptoms	matched control (healthy control)	visual stimuli - emotional faces
Voon, et al. [43]	conversion disorder (psychogenic movement disorder)	matched control (healthy control)	action selection task
Vuilleumier, et al. [22]	unilateral hysterical sensorimotor loss	within subjects design	vibratory stimulation

**Table 3:** Affected areas and sides of studies included in the meta-analysis

Citation	Area	Effect
Aybek, et al. [52]	Midbrain including periaqueductal grey area (bi), premotor and supplementary areas (bi), dorsolateral prefrontal cortex (l), cingulate cortex (l) superior frontal gyrus (l)	increased BOLD response
Aybek, et al. [47]	Supplementary motor area (r), postcentral gyrus BA1 (r), postcentral gyrus BA4/3b (r), superior temporal gyrus (r), angular gyrus at temporoparietal junction (r), supramarginal gyrus at temporoparietal junction (r)	increased BOLD response
	lingual gyrus (l), parahippocampal gyrus (l), hippocampus (l)	decreased BOLD response
Czarnecki, et al. [60]	cerebellar hemispheres (bi), superior orbital gyrus (l), inferior frontal gyrus (l), insula (l), precentral and postcentral gyri (l), supplementary motor area (r), cerebellar hemisphere and vermis (ipsi), supplementary motor area (r)	increased rCBF
	medial prefrontal cortex (bi), anterior cingulate cortex (l), cerebellum (l), lingual gyrus (l)	reduced rCBF
de Lange, et al. [16]	dorsal intraparietal sulcus (r), dorsal precentral sulcus (bi), posterior end of the Sylvian fissure	increased BOLD response to task complexity
	superior temporal cortex (l), parietal operculum, prefrontal cortex, superior temporal cortex (r), posterior end of the Sylvian fissure	increased BOLD response for the affected hand
de Lange, et al. [64]	dorsal parietal and premotor cortex	increased BOLD response to task complexity
	frontal cortex, gyrus rectus, superior temporal cortex	increased BOLD response for the affected hand
Elzinga, et al. [46]	anterior prefrontal cortex (l), dorsolateral prefrontal cortex (l), parietal lobe (l)	increased BOLD response
Spence, et al. [20]	dorsolateral prefrontal cortex	reduced rCBF
Stone, et al. [74]	basal ganglia, insula, lingual gyri, interior frontal cortex, right middle frontal gyrus (r), orbitofrontal cortex	increased BOLD response
van Beilen, et al. [50]	cingulate cortex (l), vental premotor cortex (ipsi), supramarginal cortex (ipsi), superior temporal cortex (contra + ipsi), anterior cingulate cortex (contra + ipsi), triangular cortex inferior frontal (contra)	increased BOLD response
	supramarginal gyrus (r), dlPFC (r), frontal pole (ipsi), ventral lateral prefrontal (ipsi), precuneus (contra), cerebellum (ipsi)	decreased BOLD response
Voon, et al. [43]	anterior cingulate gyrus (l), primary motor cortex (l), somatosensory cortex (l), secondary visual cortex (r), ventral premotor cortex (ipsi), supramarginal cortex (bi), anterior cingulate cortex (contra), triangular cortex (contra)	increased BOLD response
	primary motor cortex (r), somatosensory cortex (r), dorsolateral prefrontal cortex (r), medial frontal pole (r), insular cortex (l), cerebellum (l), frontal pole (ipsi), ventral lateral prefrontal (ipsi), precuneus (contra), cerebellum (ipsi), supplementary motor cortex (contra), frontal pole (contra), ventrolateral prefrontal cortex (ipsi), orbitofrontal cortex (ipsi), supramarginal cortex (contra), precuneus (contra), superior parietal cortex (contra), frontal eye fields (contra)	decreased BOLD response
Voon, et al. [27]	amygdala	increased BOLD response
	amygdala to supplementary motor area	more connectivity^a^
Vuilleumier, et al. [22]	Thalamus, caudate, putamen	decreased rCBF before treatment

*l* left, *r* right, *bi* both sides, *dlPFC* dorsolateral prefrontal cortex, *rCBF* relative cerebral blood flow, *BOLD* blood oxygen level dependent. ^a^connectivity was measured as interregional correlation between conversion tremor and voluntary tremor within the same patients

**Table 4:** Affected areas within the sample and subsamples

Analysis	Cluster	Size	Center	Gray matter at center	# of foci	Studies
all	1	456	44.7, 36.8, 26.5	BA 46: dlPFC	3	[47, 50, 74]
	2	448	19.6, −4.9, −10.5	Amygdala	4	[27, 43]
	3	248	−12.1, 51.9, 38	BA 8: superior frontal gyrus	2	[16, 64]
	4	192	−32.8, 19.7, 2	Insula	1	[16]
	5	192	−33, 39.9, 38.8	BA 8: superior frontal gyrus	2	[16, 64]
	6	176	−43.1, 34.6, 31.7	BA 9: frontal cortex	1	[46]
	7	160	−6.3, 15.3, 37.7	BA 32: dorsal ACC	1	[52]
p>c	1	544	19.7, −4.9, −10.6	Amygdala	4	[27, 43]
	2	104	−32.5, 19.1, 2.2	Insula	1	[43]
	3	80	8, −49.2, 8.8	BA 29: retrosplenial area	1	[43]
	4	72	−58.7, −48.5, 2.4	BA 22: superior temporal lobe	1	[43]
	5	72	48.9, −45.8, 10	BA 22: superior temporal lobe	1	[43]
	6	72	35.8, 50, 31.1	BA 9: frontal cortex	1	[47]
	7	64	−3, −22, .5	Red Nucleus	1	[52]
	8	64	23, 41, 5	BA 10: anterior PFC	1	[47]
	9	64	−7, −51, 7	BA 29: retrosplenial area	1	[43]
	10	64	7.5, −48.7, 21.3	BA 30: retrosplenial area	1	[43]
	11	64	47, 37, 27	BA 46: dlPFC	1	[47]
	12	64	−25, 47, 27	BA 9: frontal cortex	1	[46]
	13	64	−43, 35, 31	BA 9: frontal cortex	1	[46]
p<c	1	752	−9.4, −11.9, 69.7	BA 6: primary motor cortex	1	[43]
	2	728	−19.6, −20.2, 2	Thalamus	1	[43]
a>ua	1	1080	−32.4, 38.8, 37.8	BA 8: superior frontal gyrus	3	[16, 64]
	2	1008	−12.1, 51.6, 38.1	BA 8: superior frontal gyrus	3	[16, 64]
	3	824	7.9, 38, −12.3	BA 10: anterior PFC	3	[16, 64]
	4	680	−54.8, −11.5, 10.5	BA 43: anterior insula	2	[16, 64]
	5	168	−8.6, 13.6, 43.6	BA 32: dorsal ACC	1	[50]
	6	128	62, −29, 11.5	BA 42: dorsal temporal lobe	1	[16]
	7	128	−7.4, −40.6, 58	BA 5: primary somatosensory cortex	1	[16]
	8	128	−49.2, −34.6, −3.9	BA 21: temporal cortex	1	[16]
a<ua	1	1336	40.8, 35.4, 26.9	BA 9: frontal cortex	2	[50, 74]
	2	568	48.2, −43.3,42.4	BA 40: supramarginal gyrus	1	[50]

Size is represented in mm^3^. Clusters are described between two coordinates and its centre according to Talairach space. Each study contributing to the cluster is listed. Areas describe Brodmann areas (numbers), nearest grey matters, or nearest structures when no grey matter is within +/−5 mm. Number of underlying foci are reported for each cluster

### Meta-analysis

**Fig. 2:**
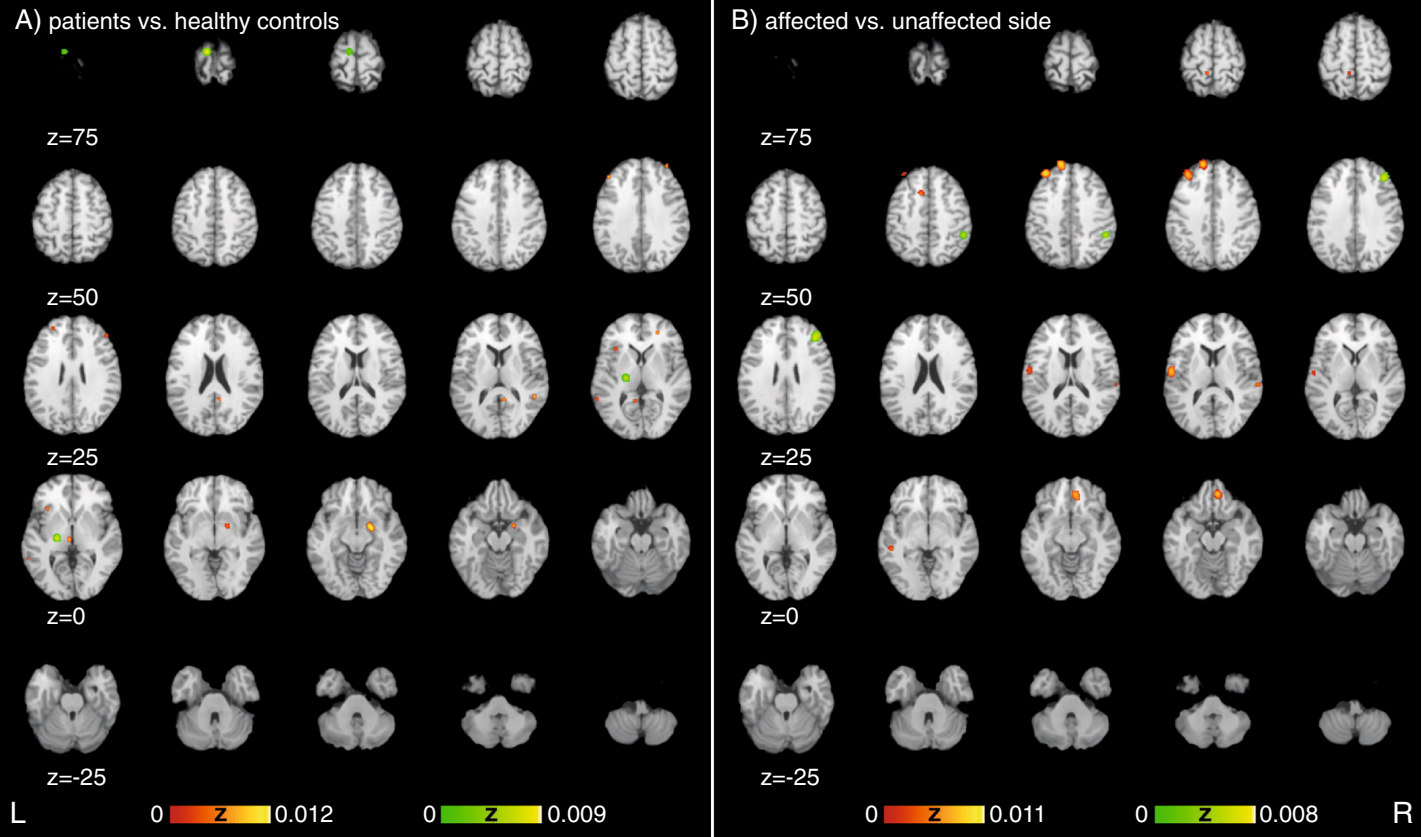
Significant areas of experiments showing differences between **a**) patients and healthy controls as well as differences between the **b**) affected versus the unaffected side sorted along the Y-axis of the Talairach space representing the dorsoventral-axis. *Red heat map* represents increased activation in patients or affected side, *green heat map* represents decreased activation in patients or affected side in comparison to control group or unaffected side respectively

**Fig. 3:**
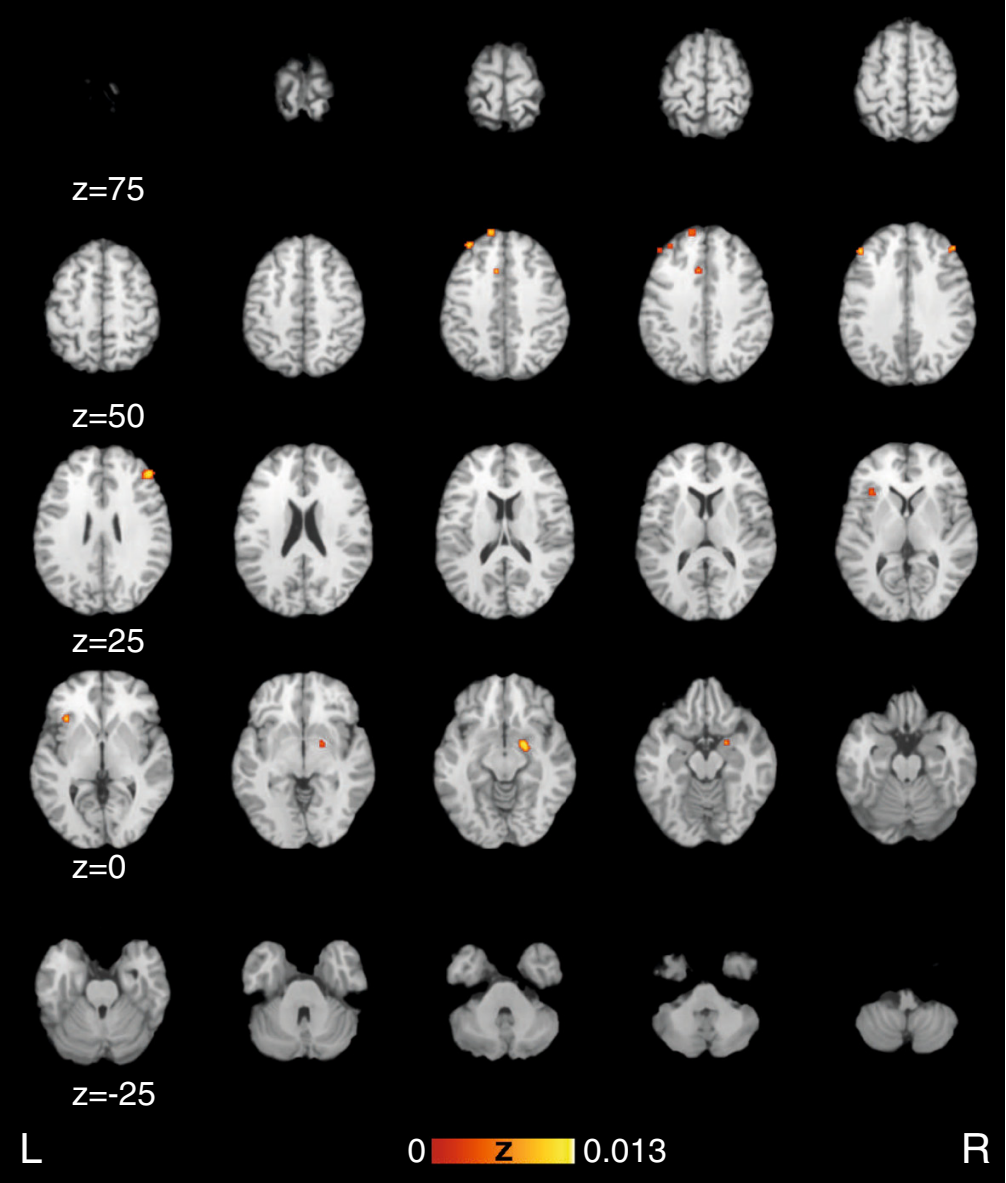
Activation likelihood estimation maps showing significant clusters of all functional experiments and overlaid on the Colin Brain. Images are sorted along the Y-axis of the Talairach space representing the dorso-ventral-axis

A cluster analysis for all experiments resulted in seven clusters (Table 4, Fig. 2), namely dorsolateral prefrontal cortex, amygdala, two clusters within the superior frontal gyrus, insula, frontal cortex as well as the dorsal anterior cingulate cortex. When calculating the subsample for p>c, 13 clusters were extracted within the following eight areas: amygdala, insula, retrosplenial area, superior temporal lobe, red nucleus, frontal cortex as well as anterior and dorsolateral prefrontal cortex (Fig. 3a). The analysis of p<c resulted in two clusters in the primary motor cortex and thalamus. Activation in the a>ua resulted in seven areas with eight clusters: superior frontal gyrus, anterior prefrontal cortex, anterior insula, dorsal anterior cingulate cortex, dorsal temporal lobe, primary somatosensory cortex, and temporal cortex (Fig. 3b). The a<ua sample shows decreased activation in the frontal cortex and the supramarginal gyrus. For details see Table 2.

## Discussion

The present study summarizes the results of functional brain-imaging (MRI, SPECT, and PET) studies on MCD via a meta-analytic approach. We found significant differential activation in several areas previously discussed in relation to CD. The current results of the meta-analysis suggest functional differences between patients with MCD and healthy controls in the amygdala, superior temporal lobe, retrosplenial area, primary motor cortex, insula, red nucleus, thalamus, anterior as well as dorsolateral prefrontal and frontal cortex (Fig. 2a). When comparing affected versus unaffected sides temporal cortex, dorsal anterior cingulate cortex, supramarginal gyrus, dorsal temporal lobe, anterior insula, primary somatosensory cortex, superior frontal gyrus and anterior as well as frontal cortex show significant differences (Fig. 2b). When analysing all functional experiments simultaneously dorsolateral prefrontal cortex, amygdala, superior frontal gyrus, insula, superior frontal gyrus, frontal cortex as well as dorsal anterior cingulate cortex show differential activation (Fig. 3).

### Patients increased activation in comparison to healthy control

The largest area of the meta-analysis comparing patients to the control population is derived from two studies [27, 43]. This cluster shows increased activity in the amygdala of patients with MCD in comparison to healthy controls. The amygdala is known to be involved in autonomic responses, including freezing behaviour, attention, vigilance and arousal [44]. Changes in this function might be an important factor for the occurrence of MCD, as patients with MCD show increased responses to startling responses [43, 45] as well as complications during the habituation to positive and negative emotional stimuli [27]. Additionally, increased functional activity in patients with MCD in comparison to healthy controls in the amygdala [27] might correlate with activity in the supplementary motor area (SMA). Voon et al. [43] suggest that the increased connectivity and activity in the SMA-amygdala motor complex facilitates the expression of previously learned conversion motor representations. This aberrant activation of prefrontal areas is also supported by our study.

Prefrontal hyper-activity are involved in clusters 6, 8, 11, 12, and 13, based on the frontal cortex (BA 9) [46, 47] anterior (BA 10) [47] as well as the dorsolateral prefrontal cortex (BA 46) [47]. Elzinga and colleagues [46] discuss the increased activation in the left anterior prefrontal cortex, left dorsolateral prefrontal cortex and left parietal lobe in correspondence to high loads and performance of the working memory in MCD patients. Aybek and colleagues [47] discuss that the increased activation in the dlPFC are based on active memory suppression during the recall of unwanted memories. Increased activation in patients with MCD was also reported during working memory studies in frontal cortex, anterior as well as dorsolateral prefrontal cortex [46]. The dorsolateral prefrontal cortex is also often dysfunctional in patients with other neuropsychiatric disorders that affect volition [20, 48, 49]. In MCD the top-down regulation of motor intention by prefrontal areas might have a crucial influence on its occurrence [43] as the decreased prefrontal activation might be correlated with impaired control of motor execution [50].

Voon et al. [43] propose the increased activation of the insula in the context of potential motor-limbic network, whereby the insula is involved in the subjective representation of internal body and feeling states during motor-selection. The increased activity of the insula found in our study might correlate with this limbic function as well as hyper-activity in the retrosplenial area, i.e. ventral posterior cingulate cortex represented in clusters 3, 9, and 10 in the analysis. This portion of the cingulate cortex is discussed to be important in the evaluation of emotional objects and memories of the past for self-relevance [43, 51].

In addition to these areas cluster 7 is centered at the red nucleus based on the study from Aybek and colleagus [52]. In their paper they do not confer the red nucleus as the most important area, but the periaqueductal grey (PAG), and hypothesize PAG to be a key region in the “freeze response” [52]. Especially the interaction between PAG and the amygdala seems to be important for autonomic fear responses and might via hyper activation result in a threat induced “freeze response” [52, 53].

The superior temporal area is another area represented in the found clusters [43]. This area incorporates the Broca area known for its contribution to language production and possibly understanding [54]. The difference between the superior temporal lobe activation in patients and controls might be based on differences in the processing of study instructions based on increased internal verbalizations of patients [55]. Additionally the temporal lobe is discussed to be a critical site in the network dealing with emotional trauma [56], whereby resolving or repressing emotional traumas seems to be partially mediated by temporal structures.

### Patients decreased activation in comparison to healthy control

Our meta-analysis shows reduced activity in the thalamus [43]. Vuilleumier et al. [22] suggest that striatothalamocortical circuits controlling voluntary motor and sensorimotor conversion are crucial for functional disorders like conversion. Hypofunction of thalamus during conversion disorder resolved after recovery [22]. This effect might be based on the function of the thalamus as the main hub system to cortical areas from sensory and motor signals; thus it plays a crucial role in generating intentional movement and learning adaptive motor action [22, 57, 58]. Reduced activity of the primary motor cortex [43] supported by our results, might correlate with the mentioned hypo-activity of the thalamus. Additionally, it might also represent reduced motor activity during motor conversion disorder.

### Affected side increased activation in comparison to unaffected side

The defect in motor action is additionally enhanced by problems with the anterior cingulate cortex (ACC; BA 32) found in cluster 5 when comparing increased activation of affected versus unaffected sides [50]. The ACC has one of its various functions in motor preparation, specifically selection of action, and conflict monitoring [59]. While Czarnecki et al. [60] report decreased activation in the ACC when comparing patients versus healthy controls, other studies report increased activation in MCD patients [43, 50, 52]. Similar to the hypothesis proposed by Voon et al. [43] in relation to increased activation of the amygdala, it might be that cingulate hyper-activation is related to emotional responses to motor action planning that inhibit motor execution especially when movement in the affected side is occurring. A study by van Beilen et al. [50] suggests that the over-activation in the cingulate cortex, especially when occurring in posterior parts, is related to alterations in functioning of the internal selection of movement that were previously described by Picard et al. [61] and might be especially pronounced when comparing movements of the affected versus unaffected side.

Increased activation of the primary somatosensory cortex [16] of the affected side might be related to increased cognitions about motor planning and sensory input. Still, especially stimulation of the affected side was associated with a decrease of the primary somatosensory cortex in sensory conversion disorder [62, 63]. Similarly to differences between patients and healthy controls, frontal and prefrontal areas are repeatedly identified within our meta-analysis when comparing increased activation in affected versus unaffected sides. The superior frontal gyrus [16, 64] and the anterior prefrontal cortex [16, 64] are significantly increased in the affected side. Increased prefrontal and frontal areas show increased activity in CP patients trying to move the affected body part [16, 17]. The increase in activity due to motor preparation of the affected side seems also associated with increased self-monitoring [16, 64–66]. Studies reporting this difference are mainly based on studies on imagination of motor initiation.

The anterior insula was significantly increased in activity when the affected side was tested [16, 64]. The region, which is continuous with the primary gustatory cortex, is involved in the experience of emotions, particularly disgust [67–69]. Additionally, it is an important integrator of multimodal stimuli responsible for interfacing internal motivational states and external information. [70–72]. Both functions seem to be increased in movement preparation in the affected compared to the unaffected side.

The dorsal temporal lobe [16] and the temporal cortex [16] are further areas showing increased activation. Similar to differences between patients and healthy controls, the temporal lobe may be involved in the network for dealing with emotional trauma, especially when resolving or repressing them [56]. The temporal region has furthermore been identified to be important for cognitive processes including implied and executed movement [73].

### Affected side decreased activation in comparison to unaffected side

Decreases in the activity of medial prefrontal cortex for the affected side have been discussed to be based on impaired willed action [50, 74]. This effect is contrary to other findings, where prefrontal activation is increased [16, 64]. This difference might be task dependent, so that some tasks like motor imagination of the affected side result in increased activation of frontal areas, while others like movement execution show decreased activation of the affected side.

The supramarginal gyrus [50] exhibited decreased activation in the affected side compared to the unaffected side. The supramarginal gyrus has been revealed to be functionally coupled in conversion disorder with the dorsolateral prefrontal cortex [21]. This connection of the prefrontal area with the sensorimotor system are involved in generating and planning motor action [75]. The decreased activation might result in abnormal movement initiation processes [50].

### All experiments

When all experiments are analysed and therefore differences between patients and healthy controls as well as differences between affected and unaffected sides are calculated within the same ALE irrespective of activation or deactivation, the following areas of interest were calculated, namely the dorsolateral and medial prefrontal cortex, the superior frontal gyrus, the insula as well as the amygdala and the dorsal anterior cingulate cortex. All areas have been identified in the sub analyses of the data. Even though no differentiation between activation or deactivation can be interpreted from the overall discussion, these areas seem to be repeatedly identified as dysfunctional in MCD and might be the core network for MCD.

### General discussion

Our results show that emotional, motor planning, and inhibitory processes are involved in MCD. Instead of single miss-functioning of a specific neuroanatomical area, a complete network of areas seems to influence the presentation of MCD symptoms. Patients with MCD seem to primarily differentiate from healthy controls in the frontal and prefrontal cortices, ACC, and amygdala relevant for motor-planning and -selection, intentional behaviour, volition, and autonomic responses. This effect, as well as the results from all included experiments, is similar to the emotional unawareness theory of Perez and colleagues [7, 33], which states that the large-scale brain network mediates emotional and cognitive mechanisms and is modulated by experience-dependent neuroplasticity. Our meta-analysis gives strong indications and supports the differences proposed in ACC, prefrontal areas, the dlPFC, and the amygdala. Our meta-analysis does not show strong evidence for changes in the posterior parietal cortex as proposed by Perez et al. [7]. In the more recent discussion of functional unawareness by Perez et al. [33] all described areas are supported by our meta-analysis. Still, strong evidence is only supported for frontal and prefrontal areas, Insula, ACC, and amygdala. In order to be able to support the suggested functional-unawareness neural circuit framework more studies have to be conducted on MCD.

In summary, our results support the perspective that specific functions, which are discussed by Perez and colleagues [7, 33] are disrupted. We found substantive backing for the disturbance of intentional capacities with our analysis based on the repeated malfunctioning of frontal and prefrontal areas [20, 21]. Changes in affective functions are highly plausible based on the increased activity of the amygdala and ACC in MCD [25–27]. Support of dysfunctional inhibitory abilities is provided by the increased activity of the affected side [16–19]. Attentional defects are partially substantiated by decreased activity of the thalamus in patients with MCD, the reduced activity of the supramarginal gyrus and increased activity of the ACC in the affected side [18, 22–24]. Alterations of action authorship in MCD is partially supported by our meta-analysis based on the changes in tempoparietal junction, somatosensory cortex, ACC, parital cortex and the temporal lobe [18, 22–24]. When considering the overall analysis, most support is provided for changes in intentional and affective functions in patients with MCD. While conversion symptoms might correlate with failures of normal neurocognitive functioning, personal experience of patients of conversion symptoms perceived as disruptive, a loss of needed information, discontinuity of experience, or recurrent, jarring, involuntary intrusions into executive functioning and sense of self should not be lost sight of [14, 76].

There is growing evidence for a relation between dissociation and trauma: on the one hand dissociative symptoms often occur in patients with PTSD [15], on the other hand depersonalization and derealisation are quintessential responses to acute trauma [14]. For the dissociative subtype of PTSD, increased activation of frontal structures is consistent with hyper inhibition of those same limbic regions in states of pathological emotional over-modulation [77]. Studies support that a PTSD dissociative subtype should be included in DSM-5 [78]. It is not surprising that a meta-analysis of neuroimaging studies of PTSD patients [15] showed an overlap in neuronal activation with the current results of MCD patients. However, some of the abnormal activations in PTSD appear to be stress related, while other activations seem to be disease related [15]. This could also apply for CD; acute dissociation is primarily related to traumatic and/or overwhelming experiences, but dissociative symptoms in life-long presentations such as Dissociative Identity Disorder may also occur in circumstances that are unrelated to trauma or overwhelming circumstances [14]. Additionally, similarities to other functional disorders might exist [79, 80]. Differences between resulting areas in the two sub-analyses for patients versus healthy controls and affected versus unaffected side might be based on the fact that we calculate inter- and intra- individual differences. Still both comparisons shed light on the development and perpetuation of the disorder.

### Limitations

This meta-analysis represents a first approach to combine the imaging results of MCD from various studies and is based on studies using differing imaging modalities and paradigms. Even though this approach might summarize various phenomena, the study of MCD is in need of a meta-analysis to more thoroughly examine the findings. The whole sample as well as the sub-analyses are sufficiently large enough to provide a first meta-analytical approach. Due to a low prevalence of MCD, the high costs of imaging, and the long data collection periods required, studies on MCD are rare. Ideally, a subcategory ALE analysis for each imaging modality and paradigm would be conducted. Due to the sparse imaging studies conducted on MCD, this was not feasible for the current study. Studies using hypnotization as a control are more prevalent, but might be explaining unintended phenomena within this meta-analysis and were therefore excluded. However, some of the mechanisms in hypnosis [81] and active feigning [19, 20] might share some neural mechanisms with MCD. Additionally, it is not clear whether the data presented in the various studies are based on recently developed conversions or on chronic conversion patients. Functional, as well as structural anatomy might change in the course of chronification. In the presented cases, not all patient characteristics are listed within the sample description, and medication as well as comorbidities might have skewed the data accordingly. Thus, it would be highly advantageous to understand functional and structural influences of the different disorders, as well as other factors influencing the variance, such as substance abuse, imaging parameters, software for analysis, experimental design etc. Future work should control for these differences, but at this time, the presented data can help present a starting point to the understanding of the neural correlates of MCD and CD in general. Still, our results are generalizable to limited extend on CD other than MCD. In order to better understand CD and specific forms of CD, more neurobiological research has to be conducted on disorders listed in Table 1, so that comparative ALEs can be calculated.

## Conclusions

With this study, we are strengthening the evidence for neurobiological factors of MCD and hope to provide a first attempt at substantiating existing explanatory models based on literature reviews alone. We are attempting to advance the understanding of the aetiology and/or maintenance of MCD, which might serve as a basis for further research on this disorder.

## Abbreviations

ALE, activation likelihood estimation; CD, conversion disorder; MCD, motor conversion disorder; PTSD, post-traumatic symptom disorder; fMRI, functional magnetic resonance tomography; VBM, voxel bases morphometry; PET, positron emission tomography; SPECT, single-photon emission computed tomography; CT, x-ray computed tomography; EEG, electroencephalography; MEG, magnetoencephalography; MNI, Montreal Neurological Institute and Hospital coordinate system; BA, Brodmann area; bi, bilateral; l, left; r, right; SMA, supplementary motor cortex; ACC, anterior cingulate cortex
